# Genetic Biodiversity and Posttranslational Modifications of Protease Serine Endopeptidase in Different Strains of *Sordaria fimicola*

**DOI:** 10.1155/2023/2088988

**Published:** 2023-02-13

**Authors:** Uzma Naureen, Ahmed Nawaz Khosa, Muhammad Azfar Mukhtar, Fazul Nabi, Nisar Ahmed, Muhammad Saleem

**Affiliations:** ^1^Molecular Genetics Research Laboratory, Department of Botany, University of the Punjab, Lahore, Pakistan; ^2^Molecular Genetics Laboratory, Lasbela University of Agriculture, Water and Marine Sciences, Uthal, Balochistan, Pakistan; ^3^Nishtar Hospital Multan, Pakistan

## Abstract

Genetic variations (mutation, crossing over, and recombination) act as a source for the gradual alternation in phenotype along a geographic transect where the environment changes. Posttranslational modifications (PTMs) predicted modifications successfully in different and the same species of living organisms. Protein diversity of living organisms is predicted by PTMs. Environmental stresses change nucleotides to produce alternations in protein structures, and these alternations have been examined through bioinformatics tools. The goal of the current study is to search the diversity of genes and posttranslational modifications of protease serine endopeptidase in various strains of *Sordaria fimicola*. The *S. fimicola*'s genomic DNA was utilized to magnify the protease serine endopeptidase (SP2) gene; the size of the product was 700 and 1400 base pairs. *Neurospora crassa* was taken as the reference strain for studying the multiple sequence alignment of the nucleotide sequence. Six polymorphic sites of six strains of *S. fimicola* with respect to *N. crassa* were under observation. Different bioinformatics tools, i.e., NetPhos 3.1, NetNES 1.1 Server, YinOYang1.2, and Mod Pred, to search phosphorylation sites, acetylation, nuclear export signals, O-glycosylation, and methylation, respectively, were used to predict PTMs. The findings of the current study were 35 phosphorylation sites on the residues of serine for protease SP2 in SFS and NFS strains of *S. fimicola* and *N. crassa*. The current study supported us to get the reality of genes involved in protease production in experimental fungi. Our study examined the genetic biodiversity in six strains of *S. fimicola* which were caused by stressful environments, and these variations are a strong reason for evolution. In this manuscript, we predicted posttranslational modifications of protease serine endopeptidase in *S. fimicola* obtained from different sites, for the first time, to see the effect of environmental stress on nucleotides, amino acids, and proteases and to study PTMs by using various bioinformatics tools. This research confirmed the genetic biodiversity and PTMs in six strains of *S. fimicola*, and the designed primers also provided strong evidence for the presence of protease serine endopeptidase in each strain of *S. fimicola*.

## 1. Introduction

After translation, proteins require chemical changes for modifications of their functions in higher-living organisms. PTMs improve the functions of proteins by conjuring up functional groups such as lipids, acetate, phosphate, and carbohydrates. The molecules of modified sites which are associated with the union and disunion of functional groups are easily explained by the fields of bioinformatics and proteomics [1]. The eukaryotic higher multicellular cells indicate PTMs (acetylation, phosphorylation, methylation, and glycosylation), because the scientific tools have approached these modifications successfully. The posttranslational modifications link with DNA, RNA, proteins, lipids, and cofactors [2]. Glycosylation, phosphorylation, acetylation, carboxylation, s-nitrosylation, and methylation are commonly researched modifications [3]. The structure of protein and its dynamic are altered by PTMs, which finally change the protein function [4, 5].

The proteins after modifications perform their duty as a factor of regulation for several cellular processes like gene expression, protein regulations, differentiation of cells, and protein degradation. The class of genus *Sordaria* is ascomycetes, and its family is sordariaceae, which is closely related to *Neurospora* sp. [6]. *Sordaria* sp. has many industrially principal enzymes like proteases, cellulases, catalases, laccases, and xylanase. Proteases have a significant role in the protein turnover process in plants throughout their life [7–9]. Protease serine endopeptidases are vital hydrolytic proteins that use the residues of catalytic serine for cleaving the peptide bonds. They can exist in all eukaryotes and prokaryotes [10]. They have a large number of cellular tasks ranging from housekeeping, i.e., signal peptide cleavage [11], immune response, protein maturation, reproduction, and apoptosis [12].

Six strains of *S. fimicola* were obtained from two slopes from Evolution Canyon (EC). This canyon, due to environmental conditions, provides a suitable platform to study the genetic variations in living organisms. Environmental stress, spontaneous mutations, recombination, and gene conversion are strong driving agents for genetic variations [13]. SFS strains of *S. fimicola* express a high rate of genetic diversity than NFS strains [4, 14]. The SFS has very dry and severe conditions, whereas NFS has gentle environmental conditions. However, environmental stress is a solid source of genetic variations [15]. The changed DNA nucleotide sequences by the genetic variations transfer into proteins and have a strong effect on posttranslational modifications. The emic research revealed a deep understanding of the relationship between genetic diversity, environmental factors, and metabolites' concentration [16]. As far as we know, no study on PTMs of protease serine endopeptidase in the experimental fungus has been conducted so far. Therefore, the current study was focused on the modifications of protease serine endopeptidase such as glycosylation, acetylation, phosphorylation, and methylation in experimental and reference fungi.

## 2. Methodology

### 2.1. The Subculturing of Experimental Fungal Strains

The NFS and SFS strains of *S. fimicola* were provided by the Molecular Genetics Research Laboratory of Botany Department, PU, Lahore. The experiments were performed at Molecular Genetics Laboratory, Faculty of Veterinary and Animal Sciences, Lasbela University of Agriculture, Water and Marine Sciences, Uthal, Balochistan, and Molecular Genetics Research Laboratory, Department of Botany, University of the Punjab, Lahore, Pakistan. The subculturing was done from the stock of fungus (preserved at -20°C) which was initially obtained from “Evolutionary Canyon” by Prof. Nevo. Different strains of *S. fimicola* had been obtained from three different places on the north-facing slope (NFS) and called N5, N6, and N7, whereas the S strains had been collected from three stations of the south-facing slope (SFS), and these were called S1, S2, and S3. *S. fimicola* strains were subcultured on growth media, i.e., PDA (Potato Dextrose Agar), under sterilized conditions to keep away any contamination and kept in an incubator at 20°C to get perithecium of *S. fimicola* within 10-12 days for DNA extraction.

### 2.2. Genomic DNA Extraction

*S. fimicola* strains were used to extract genomic DNA by following the protocol performed by [17]. The isolated DNA was utilized to 0.8% agarose for gel electrophoreses which was dyed with ethidium bromide with 1-kilobase DNA ladder and bands examined under gel docs (documentation system (U:Genius3-Syngene)). The designing of primers and their concentration's preparation were done. The primers were designed for the amplification of protease serine endopeptidase gene by the NCBI Primer Blast and Primer 3 Plus tool. In a current study, two pairs of primers were used (Table 1). Then, the primers' stock concentrations (100 *μ*M) were prepared, and working stock of 10 *μ*M of each primer was also prepared and stored at -20°C. The amplification of targeted protease serine endopeptidase genes was done through PCR. The reaction volume of PCR was made up to 30 *μ*l, which contained 15 *μ*l PCR master mix, 2.5 *μ*l fungal genomic DNA, 1.50 *μ*l for each forward and reverse primer, 1.50 *μ*l of 25 mM MgCl_2_, and 9 *μ*l of double-distilled H_2_O. The first round of amplification consists of initial denaturation at 95°C for 10 min followed by 30 cycles of denaturation at 95°C for 25 sec, annealing at 56°C for 45 sec, and extension at 72°C for 1 min, with a final extension step of 72°C for 8 min. The PCR products were run on gel electrophoreses on 0.8% agarose gel for the confirmation of amplification. Then, gene sequencing of the PCR products was achieved. The eluted bands were sequenced. Online server EMBOSS Transeq (https://www.ebi.ac.uk/Tools/st/emboss_transeq/) was used to obtain the protein sequence of genes. The nucleotide sequence of the reference organism protease serine peptidase gene was obtained from NCBI (http://blast.ncbi.nlm.nih.gov/Blast). The analysis of sequenced data Clustal Omega (http://ebi.ac.uk/Tools/msa/clustalo/) was used to check the nucleotide and amino acid variations in gene and protein sequences of different strains of experimental and reference fungi. The designed primers confirmed the presence of dipeptidyl-aminopeptidase protease in *S. fimicola*.

### 2.3. PTMs: Prediction Tools

Different bioinformatics tools for PTM prediction were used. Phosphorylation with NetPhos 3.1, glycosylation with YinOYang 1.2, methylation with Mod Pred, and NES (nuclear export signals) with NetNES 1.1 servers were predicted. EMBOSS Transeq, an online tool, was used for the amino acid sequences of amplified SP2 genes, whereas amino acid sequences of the reference strain were recovered from Uniprot.

### 2.4. Pymol: Prediction Tool

A prediction tool, Pymol, was used to form 3D models of protease SP2 with a high degree of confidence. This prediction tool is reliable.

## 3. Results

### 3.1. Multiple Sequence Alignment

**Figure 1:**
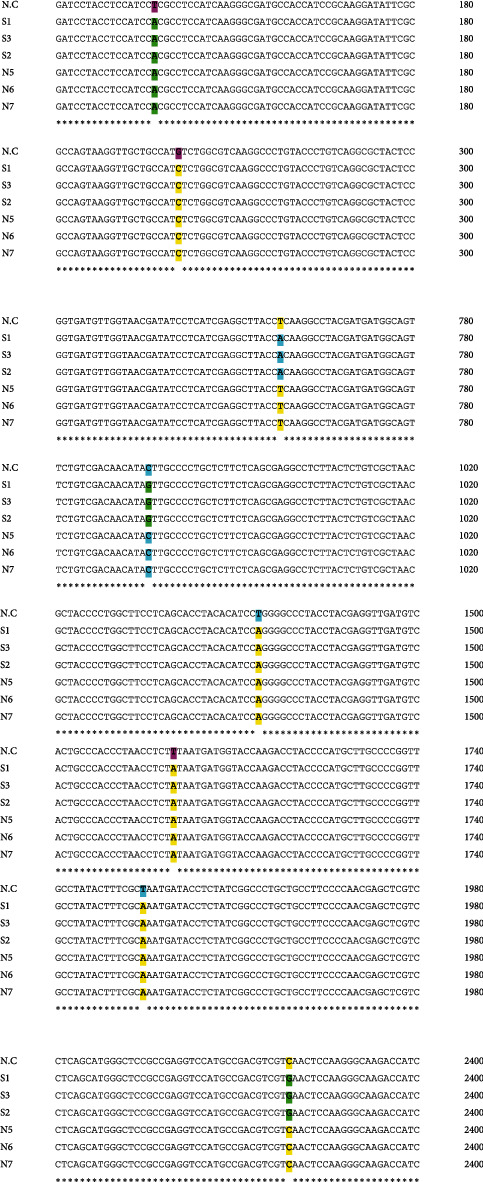
Nucleotide sequence alignment of *Sordaria fimicola* strains with reference fungus. Polymorphic sites are shown by the gaps.

The extracted DNA of *S. fimicola* strains was utilized for the amplification of the protease serine endopeptidase gene. Clustal Omega software was used for the study of polymorphism by aligning the nucleotide sequence of different strains of experimental and reference fungi. Eight polymorphic sites are observed in the SP2 gene of *S. fimicola* strains compared to the reference fungus (Figure 1). In the 1^st^ polymorphic site, at the 137^th^ position (121-180) of the reference fungus, “thymine” is changed with adenine in the experimental fungus (NFS, SFS), resulting in the change of CTC codon into CAC, which finally changed the amino acid leucine (Leu) into histidine (His). In the 2^nd^ polymorphic site, at position 261 (241-300) of the reference fungus, “guanine” is replaced with “cytosine” in the experimental fungus (NFS, SFS); TGT codon is altered into TCT, which ultimately changed the amino acid cysteine (Cys) into serine (Ser). In the 3^rd^ polymorphic site, at position 757 (721-780) of the reference fungus and NFS strains of the experimental fungus, “thymine” is replaced with “adenine” in SFS strains of the experimental fungus, resulting in CTC codon being altered into CAC, which ultimately changed leucine (Leu) into histidine (His). In the 4^th^ polymorphic site, at position 977 (960-1020) of the reference fungus and NFS strains of the experimental fungus, “cytosine” is replaced with “guanine” in SFS strains of the experimental fungus, resulting in ACT codon being altered into AGT, which ultimately changed threonine (Thr) into serine (Ser). In the 5^th^ polymorphic site, at position 1470 (1441-1500) of the reference fungus, “thymine” is replaced with “adenine” of SFS and NSF strains of *S. fimicola*, resulting in CTG codon being altered into GAG, which ultimately changed leucine (Leu) into glutamine (Gln). In the 6^th^ polymorphic site, at position 1700 (1681-1740) of the reference fungus, thymine is replaced with “adenine” in SFS and NSF strains of the experimental fungus, resulting in TTT codon being altered into TAT, which ultimately changed phenylalanine (Phe) into tyrosine (Tyr). In the 7^th^ polymorphic site, at position 1938 (1921-1980) of the reference fungus, “thymine” is replaced with “adenine” in NSF and SFS strains of the experimental fungus, resulting in CTA codon being altered into CAA, which ultimately changed leucine (Leu) into proline (Pr). In the 8^th^ polymorphic site, at position 2341-2400 of the reference fungus, “cytosine” is replaced with “guanine” in SFS and NFS of the experimental fungus, resulting in a change in TCA codon into TGA, which ultimately changed serine (Ser) into stop codon (Figure 1).

**Figure 2:**
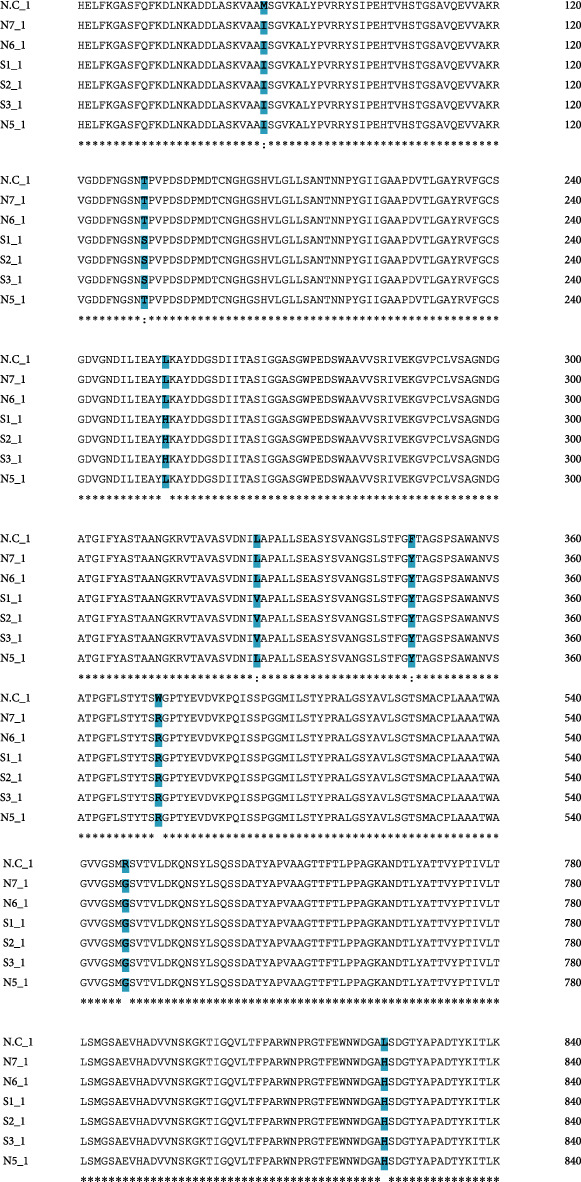
Amino acid sequence alignment of *Sordaria fimicola* strains with reference fungus. The polymorphic sites are shown by gaps, and conservation among the species of strongly similar properties is represented by (:).

The sequences were used in the Blast tool at NCBI to check homologous sequences in the experimental fungus with reference fungus. Amino acid sequence alignment of SFS and NFS strains of the experimental fungus with the reference fungus has shown a total of 8 polymorphic sites (Figure 2).

### 3.2. O-Glycosylation and YinOYang: Prediction Sites

**Figure 3:**
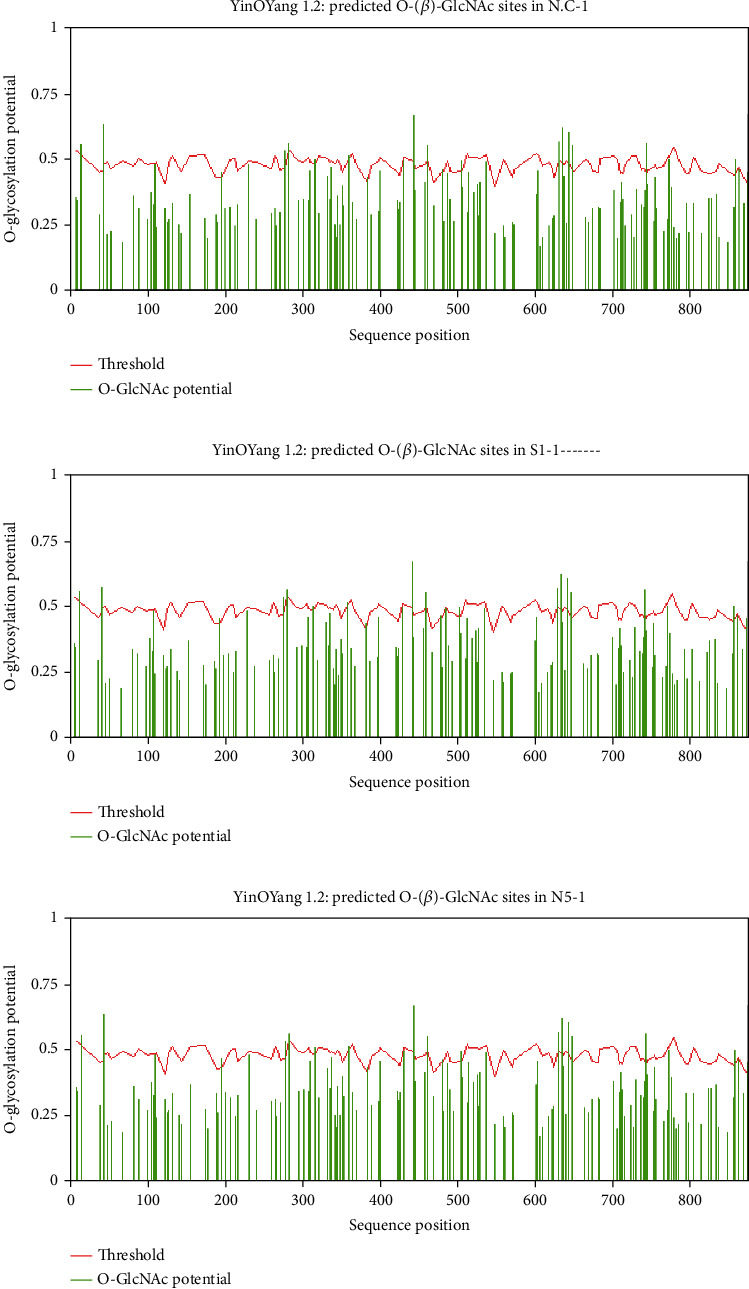
Glycosylation potential graphs of modified sites of O-linked acetyl glucosamine N. crassa, SFS strains, and NFS strains of S. fimicola (a–c), respectively. Green-colored lines show O-GlcNAc potential; red horizontal line shows the threshold level (0.5).

The predicted sites of YinOYang and O-glycosylation at residues of threonine and serine for serine endopeptidase proteases of six strains of experimental and reference fungi were obtained by YinOYang 1.2 and are presented in Table 2. The findings of the YinOYang 1.2 server explained that glycosylation occurred on serine residue at seven positions, i.e., 15, 44, 277, 283, 360, 649, and 743, whereas on residues of threonine, glycosylation is observed at five positions, i.e., 443, 461, 630, 636, and 643 in *N. crassa*. There is glycosylation on serine residue at eight positions, i.e., 15, 44, 277, 283, 360, 505, 649, and 743, whereas glycosylation on the threonine residue is at six positions, i.e., 317, 443, 461, 630, 636, and 643, in *S. fimicola* (Table 2). A graphical representation of glycosylation in SP2 of six strains of experimental and reference fungi is shown in Figure 3. The sites of acetylation were observed at positions 50, 65, 72, 76, 83, 91, 119, 146, 149, 254, 314, 404, 411, 414, 427, 437, 545, and 649 on K (internal lysine) in strains of experimental and reference fungi (Table 2).

### 3.3. Phosphorylation and NetPhos 3.1's Prediction Sites

The results of the NetPhos 3.1 server revealed that phosphorylation occurred on protease serine endopeptidase of reference fungus and six experimental fungal strains on the following residues, i.e., tyrosine (Y), threonine (T), and serine (S). The predicted sites on serine were S-6, S-39, S-44, S-48, S-82, S-88, S-100, S-108, S-128, S-141, S-195, S-240, S-266, S-308, S-332, S-335, S-342, S-344, S-352, S-425, S-431, S-491, S-606, S-742, and S-848; on threonine, 4, 144, 159, 195, 264, 345, 398, 426, 488, 548, 610, 624, 665, 682, 710, 772, 799, 815, 834, and 858; and on tyrosine, 36, 220, 336, 415, 642, 715, 747, and 829, observed in the experimental fungus and *N. crassa* in Table 3.

### 3.4. Methylation and Mod Pred's Prediction Sites

In the reference fungus, the methylation residues of lysine (83, 389, 414, 427, 680, 734, 796, 798, 869, and 871) were investigated, whereas the following four methylation residues at arginine (3, 517, 809, and 872) and 12 lysine residues (83, 314, 389, 414, 427, 680, 734, 796, 798, 836, 869, and 871) were seen in six strains of the experimental fungus in Table 4.

### 3.5. Nuclear Export Signals (NES): Prediction Sites

**Figure 4:**
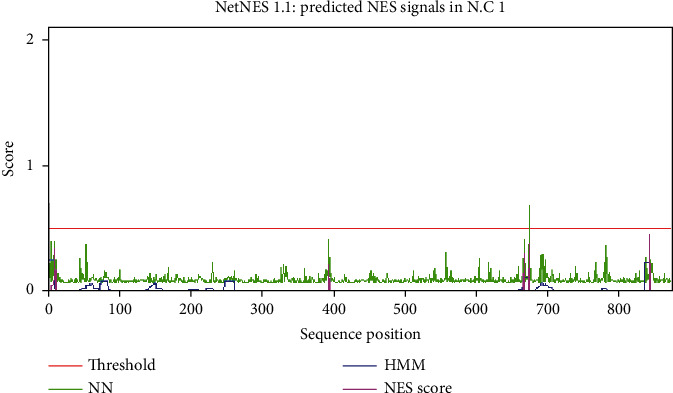
Graph of nuclear export signals (leucine-rich) of *N. crassa*. NN signals are represented in green peaks, HMM signals are represented in blue peaks, and NES signals are represented in purple peaks. The threshold level is shown in the red line (0.5).

**Figure 5:**
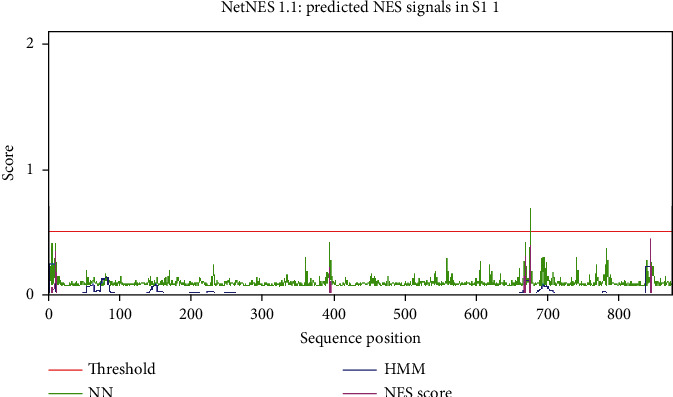
Graph of nuclear export signals (leucine-rich) of *S. fimicola*. NN signals are represented in green peaks, HMM signals are represented in blue peaks, and NES signals are represented in purple peaks. The threshold level is shown in the red line (0.5).

In the current study, 675-L, 844-I, and 394-I are three NES of protease SP2 in the reference fungus, whereas in south-facing slope strains of the experimental fungus, 675-L, 688-L, 844-I, and 675-L; 844-I, 11-L, and 10-L positions were observed in the north-facing slope of the experimental fungus. Two sites 675-L and 844-I are highly conserved in the experimental fungus as shown in Table 3 and Figures 4 and 5.

### 3.6. Pymol for 3D Protein Structure

**Figure 6:**
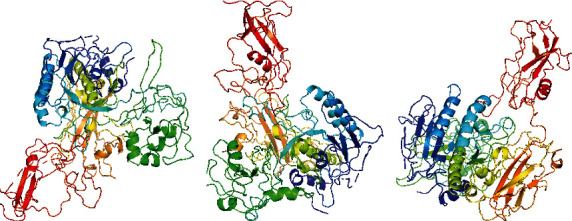
Pymol-3D structures of SP2 *N. crassa* (a), S2 (b), and N6 (c).

The SP2 proteins of two strains of S2 and N6 of experimental and reference fungi were seen by Pymol (Figure 6). The coil structures are represented by sticks, and *β*-sheets are represented by arrows, whereas *α*-helix is shown by sticks and cartoons. The dimensions of the reference fungus are (Å) *X*: 73.428, *Y*: 97.011, and *Z*: 75.603 as shown in Figure 6(a). The dimensions of S2 strain of *S. fimicola* (Å): *X*: 70.248, *Y*: 90.689, and *Z*: 75.603 as in Figure 6(b) and of N6 strain of *S. fimicola* (Å): *X*: 70.248, *Y*: 97.011, and *Z*: 75.603 as in Figure 6(c).

## 4. Discussion

According to the information, the protease serine endopeptidase gene is examined for the first time in the experimental fungus. In the present research, variations of genes and PTMs of protease serine peptidase of different strains of *S. fimicola* were under observation. *S. fimicola*'s SFS and NFS strains showed 8 polymorphism sites (Figure 1). Protease serine peptidase regions have 9 sites of nonsynonymous substitutions (Figure 2). The south-facing slope (SFS) has xeric and stressful environmental conditions, so it exhibited more genetic variations as compared to the north-facing slope. Closely related organisms have more tendency of polymorphism in specific locations of their DNA [18]. *Aspergillus niger* and *Penicillium* sp. showed dominant polymorphism because Evolution Canyon 1 has a stressful environment which suggested that genetic diversity (polymorphism) is caused by a stressful environment. The biodiversity of genes of *S. fimicola* from Evolution Canyon was reported by [13, 19]. They said that genetic biodiversity is also caused by gene conversion and mutation. They concluded that *S. fimicola* strains of SFS had a high rate of mutation due to crossing over and spontaneous mutations. Natural selection of living organisms occurs by climatic conditions, and as a result, parental and genetic variants are produced. Evolution is caused by these variations, and these variations also originate in the evolutionary potential of living organisms. [4, 20] described frequency clock and mating type a-1 proteins of different strains of *S. fimicola* obtained from “Evolution Canyon,” Israel, where genetic variations have strong effects on PTMs of proteins. Hence, the polymorphism in the positions of protease serine endopeptidase in six *S. fimicola* strains is due to the strong impact of genetic variations (Tables 1–3). Genetic variations change DNA nucleotide sequences, and these nucleotide sequences produce proteins of unique PTM sites. After translation, these PTMs are responsible for protein diversity. Posttranslational modifications alter protein configuration. Furthermore, it is a necessity for scientific research to study PTMs and understand how these PTMs work to carry on the function of proteins [21]. The PTMs like glycosylation, acetylation, phosphorylation, methylation, and carboxylation are commonly studied in living organisms [22]. In the current study, glycosylation, phosphorylation, acetylation, and methylation of SP2 of *Sordaria* sp. were mainly examined.

Protein phosphorylation regulates biological processes. Phosphorylation is categorized by the reversible enzymatic addition of a phosphate group to amino acid side chains of serine (Ser), threonine (Thr), or tyrosine (Tyr), which alter the structure and stability of proteins [23]. The role of phosphorylation is significant in conformational changes like activation, deactivation of proteins, and specificity of binding [24, 25].

Our work observed 27 sites of phosphorylation on serine residues for SP2 in the experimental fungus and *N. crassa* (Table 2). In most of the eukaryotes, the phosphorylation events occur at specific serine residues which has effects of signaling [6]. The current research revealed that the following sites of phosphorylation on serine (S-39, S-141, and S-352) were conserved in experimental and reference fungi. Previously, three phosphorylation sites (S-32, S-160, and S-365) were predicted which have been confirmed by mass spectrometry in matrix metalloproteinases (MMP2) [26, 27]. In multicellular organisms, the role of Ser-248 was conserved in cell development control [28]. The threonine phosphorylation modifications play a vital role in the regulation of the functions of cells. The current study depicted 20 phosphorylation sites on threonine residues for protease serine endopeptidase in six strains of *S. fimicola* and *N. crassa* (Table 2). Among 20 phosphorylation sites on threonine, some sites (T-195, T-548, and T-624) were predicted whereas T-567 phosphorylation promotes MMP14 (matrix metalloproteinases), which induced the cellular invasion and migration [29–33]. Moreover, it is reported that the phosphorylation at tyrosine causes cell signaling [34]. The current exploration investigated seven sites of phosphorylation on tyrosine residues for protease serine endopeptidase in six strains of *S. fimicola* and reference fungus (Table 1). Some sites (Y-220, Y-336, and Y-715) were investigated among 7 phosphorylation sites on tyrosine. Protein kinases play a very vital role in phosphorylation. The main task of these kinases is to transfer a phosphate group from ATP to the substrate and phosphorylate it. CDC2, CK2, UNSP, PKC, PKA, and DNA-PK are involved in phosphorylation for SP2 in experimental and reference fungi. Phosphorylation of cytochrome C oxidase (COX1) is carried out by PKA, PKC, CDC2, and UNSP [4]. The 19, 15, and 17 phosphorylation sites on serine residues for CpA1 (protease carboxypeptidase A1) in S1, N7 (strains of *S. fimicola*), and *N. crassa*, respectively, were reported by [35]. The PKA, PKC, CKII, and UNSP are actively involved in the phosphorylation of RKM4 protein of *Sordaria* species. The phosphorylation within a cell is carried out by protein kinase CK2. ATP or GTP is a major source of phosphate. In eukaryote DUBA deubiquitinating movement, CK2 kinases are involved in the phosphorylation of Ser 77. However, CK2 phosphorylation does not involve any organizational variation. The phosphate group reduces the substrate-protease collaboration but does not involve the active site [36, 37].

Acetylation of lysine is a dynamic and reversible PTM in proteins in eukaryotes [38–42]. However, 50% acetylated proteins are present in *Sordaria* sp., and some observed acetylation sites on internal lysine (K) in the current study are 50, 65, 72, 76, 83, 91, 119, 146, 149, 254, 427, 545, and 649 in the experimental fungus and reference strain (Table 1). There are 16 acetylation sites of CpA1 in *S. fimicola* and 14 in *N. crassa* [35]. COX1 (cytochrome C oxidase-1) protein of *Sordaria sp*. has a lysine site at 437, and in the current study, the lysine site at 437 is also investigated in SP2 in the experimental fungus [43]. Besides this, the lysine site is present at 119 in CyC-1 (cytochrome C-1) of different strains of *Sordaria sp.* [44], whereas in the current study, this site is also present in the SP2 gene in the experimental fungus. All these predictions show the conservation of the site, and this site also actively participated in the regulation of SP2, COX1, and CyC-1.

In glycosylation, proteins and lipids are linked with saccharides by enzymes. N- and O-linked glycosylation is very common [45–47]. Protein glycosylation played a vital role in cell adhesion, protein folding, and tracking inside and outside the cell. Diabetes, carcinoma, and brain diseases are linked with changes in the glycosylation pattern [48, 49]. In a current study, 12 sites of O-glycosylation for SP2 were investigated in the experimental organism and 13 sites in the reference fungus. T-461 is the threonine glycosylation site, whereas the T-460 site is for MMP14 (matrix metalloproteinases) as observed by [29, 50, 51]. Interestingly, two novel sites (S-505^∗^ and T-317^∗^) were examined in *S. fimicola*, but these are absent in the reference fungus (Table 1). All glycosylation sites are shown in Figure 3.

Methylation of proteins is an important reversible PTM. The researchers examined N-methylations of lysine and arginine residues due to their significance [52, 53]. Ascomycetes have many methylation sites. Methyltransferases are cordially engaged in biological pathways [54, 55]. The current study predicted 4 residues of arginine of methylation (R-3, R-517, R-809, and R-872), which are highly conserved for experimental and reference fungi. The following residues of lysine (K-83, K-389, K-414, K-427, K-680, K-734, K-796, K-798, K-869, and K-871) of methylation are highly conserved for experimental and reference fungi. The K-314 and K-836 (methylated sites) are involved only in the experimental fungus.

There are many pathways of nuclear export signals, and leucine-rich is the best one [56]. The nuclear export signals were studied in man immunodeficiency virus type-1 rev protein reported for the first time [57] and investigated in cAMP-dependent protein kinase inhibitor. The subcellular localization of the molecules of organisms is regulated by nuclear export signals [58]. Nuclear export signals help the factors and protein interactions to leave the cytoplasm [59]. In this study, the prediction of these signals is at the 675-L position of SP2 in *S. fimicola* and position 675-L in the same protein of the reference fungus, which is an indication of this protein through NES (Figures 4 and 5). The prediction of NES at position 328 of frequency clock protein in *S. fimicola* and position 323 in *N. crassa* is an indication of protein regulation through nuclear export signals [4].

## 5. Conclusion

This study suggested that genetic biodiversity and posttranslational modifications in six strains of *S. fimicola* tend to bear environmental stress which may be helpful in various industries such as brewing, detergent, leather, dairy, and food processing factory. The current study suggested the production of fungal proteases commercially.

## Figures and Tables

**Table 1:** Primers for experimental fungus.

Primer sets of protease	Forward primer sequence (5′-3′)	Reverse primer sequence (5′-3′)	Region targeted	Primer source
SP2				
Pair 1	ATGGTTCGCTTCGGCCTCGCC	GGCACCACCACCGATGGAGGCGGT	Serine endopeptidase	Self-designed
Pair 2	GGGTATCTTCTACGCTTCTAC	CCGCTTGGACGTAAAGCCCTCTTACTCTT	Serine endopeptidase	Self-designed

**Table 2:** Sites of O-glycosylation and phosphorylation for SP2 of experimental and reference fungi.

Organism	Amino acid residues	Glycosylation positions	Acetylation on lysine (K)
*N. crassa*	S	15, 44, 277, 283, 360, 649, 743 (07)	50, 65, 72, 76, 83, 91, 119, 146, 149, 254, 314, 404, 411, 414, 427, 437, 545, 649 (18)
	T	443, 461, 630, 636, 643 (05)	
	Y	—	

SFS strains	S	15, 44, 277, 283, 360, 505∗, 649, 743 (08)	50, 65, 72, 76, 83, 91, 119, 146, 149, 254, 314, 404, 411, 414, 427, 437, 545, 649 (18)
	T	317∗, 443, 461, 630, 636, 643 (06)	
	Y	—	

NFS strains	S	15, 44, 277, 283, 360, 505∗, 649, 743 (08)	50, 65, 72, 76, 83, 91, 119, 146, 149, 254, 314, 404, 411, 414, 427, 437, 545, 649 (18)
	T	317∗, 443, 461, 630, 636, 643 (06)	
	Y	—	

**Table 3:** Sites of phosphorylation with their protein kinases for SP2 of experimental and reference fungi.

Organisms	Residues	Phosphorylation sites	Protein kinases					
			CDC2	CKII	UNSP	PKC	PKA	DNAPK
*N. crassa*	Serine (S)	6, 39, 48, 82, 100, 108, 141, 195, 240, 266, 308, 332, 335, 342, 344, 352, 425, 491, 606, 716, 725, 740, 742, 765, 785, 825, 848, 870 Total = 27	335, 337 352	39, 195 240, 825	6, 44, 100, 108, 141, 352425, 491, 606, 740 742, 785, 870	82, 108, 266 308, 332, 344, 425, 601, 725 765, 870	100 141 716 848	342 740
	Threonine (T)	4, 144, 159, 195, 264, 345, 398, 426, 488, 548, 610, 624, 665, 682, 710, 772, 799, 815, 838, 858 Total = 20	264, 345 514, 710 772	610	4, 159, 195 398, 426, 548, 624, 772, 815 834, 858	144, 345 398, 488, 548 624, 815, 838	398 665 682 799	458 828 856
	Tyrosine (Y)	36, 220, 336, 642, 715, 747, 829 Total = 7	—	—	36, 220, 336 642, 715, 747, 829	—	—	—

*S. fimicola* (SFS, NSF)	Serine (S)	6, 39, 48, 82, 100, 108, 141, 195, 240, 266, 308, 332, 335, 342, 344, 352, 425, 491, 606, 716, 725, 740, 742, 765, 785, 825, 848, 870 Total = 27	335, 337 352	39, 195 240, 825	6, 44, 100, 108, 141, 352425, 491, 606, 740 742, 785, 870	82, 108, 266 308, 332, 344, 425, 601, 725 765, 870	100 141 716 848	342 740
	Threonine (T)	4, 144, 159, 195, 264, 345, 398, 426, 488, 548, 610, 624, 665, 682, 710, 772, 799, 815, 838, 858 Total = 20	264, 345 514, 710 772	610	4, 159, 195 398, 426, 548, 624, 772, 815 834, 858	144, 345 398, 488, 548 624, 815, 838	398 665 682 799	458 828 856
	Tyrosine (Y)	36, 220, 336, 642, 715, 747, 829 Total = 7	—	—	36, 220, 336, 642, 715, 747, 829	—	—	—

**Table 4:** Sites of methylation and nuclear export signals for SP2 gene of experimental fungus and reference fungus.

Server	Fungi	Amino acid residues	Sites (methylation)	Server	Sites (NES)	Nuclear export signals
Mod Pred	*N. crassa*	R (arginine)	3, 517, 809, 872 (total = 4)		675-L	0.674
		K (lysine)	83, 389, 414, 427, 680, 734, 796, 798, 869, 871 Total = 10		844-I 394-I	0.421 0.408
	*S. fimicola* (SFS) S1, S2, S3	Arginine (R)	3, 512, 809, 872 Total = 4	NetNES 1.1	675-L 844-I	0.674 0.421
		Lysine (K)	83, 314, 389, 414, 427, 680, 734, 796, 798, 836, 869, 781 Total = 12		668-L	0.412
	*S. fimicola* (NFS) N5, N6, N7	Arginine (R)	3, 517, 809, 872 Total = 4		675-L 844-I	0.674 0.421
		Lysine (K)	83, 389, 414, 427, 680, 734, 796, 798, 836, 869, 871 Total = 11		11-L 10-L	0.403 0.400

## Data Availability

The data used to support the findings of this study are available from the corresponding author upon request.

## References

[1] Chandramouli K., Qian P.-Y. (2009). Proteomics: challenges, techniques and possibilities to overcome biological sample complexity. *Human genomics and proteomics: HGP*.

[2] Sacco F., Perfetto L., Castagnoli L., Cesareni G. (2012). The human phosphatase interactome: an intricate family portrait. *FEBS Letters*.

[3] Marquez J., Lee S. R., Kim N., Han J. (2016). Post-translational modifications of cardiac mitochondrial proteins in cardiovascular disease: not lost in translation. *Korean Circulation Journal*.

[4] Arif R., Akram F., Jamil T., Mukhtar H., Lee S. F., Saleem M. (2017). Genetic Variation and Its Reflection on Posttranslational Modifications in Frequency Clock and Mating Type a-1 Proteins in *Sordaria fimicola*. *BioMed Research International*.

[5] Bhadauria V., Zhao W.-S., Wang L.-X. (2007). Advances in fungal proteomics. *Microbiological Research*.

[6] Ishfaq M., Mahmood N., Nasir I. A., Saleem M. (2017). Biochemical and molecular analysis of superoxide dismutase in Sordaria fimicola and Aspergillus niger collected from different environments. *Polish Journal of Environmental Studies*.

[7] Panicker L. M., Usha R., Roy S., Mandal C. (2009). Purification and characterization of a serine protease (CESP) from mature coconut endosperm. *BMC Research Notes*.

[8] Müntz K., Belozersky M., Dunaevsky Y., Schlereth A., Tiedemann J. (2001). Stored proteinases and the initiation of storage protein mobilization in seeds during germination and seedling growth. *Journal of Experimental Botany*.

[9] Fontanini D., Jones B. L. (2002). SEP-1 - a subtilisin-like serine endopeptidase from germinated seeds of Hordeum vulgare L. cv. Morex. *Planta*.

[10] Page M. J., Di Cera E. (2008). Serine peptidases: classification, structure and function. *Cellular and Molecular Life Sciences*.

[11] Gabriely G., Kama R., Gerst J. E. (2007). Involvement of specific COPI subunits in protein sorting from the late endosome to the vacuole in yeast. *Molecular and Cellular Biology*.

[12] Hedstrom L. (2002). Serine protease mechanism and specificity. *Chemical Reviews*.

[13] Saleem M., Lamb B. C., Nevo E. (2001). Inherited differences in crossing over and gene conversion frequencies between wild strains of Sordaria fimicola from “Evolution Canyon”. *Genetics*.

[14] Jamil T., Ijaz S., Arif R., Akram F., Saleem M. (2019). Diversity of retrotransposons (Ty3-gypsy, LINEs and Ty1-copia) in Sordaria fimicola. *Pakistan Journal of Zoology*.

[15] Nevo E. (2012). “Evolution Canyon,” a potential microscale monitor of global warming across life. *Proceedings of the National Academy of Sciences*.

[16] Téllez-Téllez M., Diaz-Godinez G. (2019). Omic tools to study enzyme production from fungi in the Pleurotus genus. *BioResources*.

[17] Pietro S., Fulton T., Chunwongesm J., Tanksley S. (1995). Microprep protocol for Extraction of DNA from tomato and other herbaceous plants. *Molecular Biology Report*.

[18] Park E.-J., Fukuda S., Endo H., Kitade Y., Saga N. (2007). Genetic polymorphism withinPorphyra yezoensis(Bangiales, Rhodophyta) and related species from Japan and Korea detected by cleaved amplified polymorphic sequence analysis. *European Journal of Phycology*.

[19] Lamb B. C., Mandaokar S., Bahsoun B., Grishkan I., Nevo E. (2008). Differences in spontaneous mutation frequencies as a function of environmental stress in soil fungi at “Evolution Canyon,” Israel. *Proceedings of the National Academy of Sciences*.

[20] Arber W. (2000). Genetic variation: molecular mechanisms and impact on microbial evolution. *FEMS Microbiology Reviews*.

[21] Lothrop A. P., Torres M. P., Fuchs S. M. (2013). Deciphering post-translational modification codes. *FEBS Letters*.

[22] Oliveira A. P., Sauer U. (2012). The importance of post-translational modifications in regulating Saccharomyces cerevisiae metabolism. *FEMS Yeast Research*.

[23] Ardito F., Giuliani M., Perrone D., Troiano G., Lo Muzio L. (2017). The crucial role of protein phosphorylation in cell signaling and its use as targeted therapy (review). *International Journal of Molecular Medicine*.

[24] Cohen P. (2001). The role of protein phosphorylation in human health and disease. *European Journal of Biochemistry*.

[25] Cutillas P. (2017). Targeted in-depth quantification of signaling using label-free mass spectrometry. *Methods in Enzymology*.

[26] Sariahmetoglu M., Crawford B. D., Leon H. (2007). Regulation of matrix metalloproteinase-2 (MMP-2) activity by phosphorylation. *The FASEB Journal*.

[27] Jacob-Ferreira A. L., Kondo M. Y., Baral P. K. (2013). Phosphorylation status of 72 kDa MMP-2 determines its structure and activity in response to peroxynitrite. *PLoS One*.

[28] Horn D. L., Neofytos D., Anaissie E. J. (2009). Epidemiology and outcomes of candidemia in 2019 patients: data from the prospective antifungal therapy alliance registry. *Clinical Infectious Diseases*.

[29] García-Pardo A., Opdenakker G. (2015). Nonproteolytic functions of matrix metalloproteinases in pathology and insights for the development of novel therapeutic inhibitors. *Metalloproteinases Med*.

[30] Williams K. C., Coppolino M. G. (2011). Phosphorylation of membrane type 1-matrix metalloproteinase (MT1-MMP) and its vesicle-associated membrane protein 7 (VAMP7)-dependent trafficking facilitate cell invasion and migration. *Journal of Biological Chemistry*.

[31] Moss N. M., Wu Y. I., Liu Y., Munshi H., Stack M. S. (2009). Modulation of the Membrane Type 1 Matrix Metalloproteinase Cytoplasmic Tail Enhances Tumor Cell Invasion and Proliferation in Three-dimensional Collagen Matrices. *Journal of Biological Chemistry*.

[32] Bordoli M. R., Yum J., Breitkopf S. B. (2014). A secreted tyrosine kinase acts in the extracellular environment. *Cell*.

[33] Nyalendo C., Michaud M., Beaulieu E. (2007). Src-dependent phosphorylation of membrane type i matrix metalloproteinase on cytoplasmic tyrosine 573:. *Journal of Biological Chemistry*.

[34] Ishfaq M., Mahmood N., Nasir I. A., Saleem M. (2014). Molecular and biochemical screening of local Aspergillus niger strains efficient in catalase and laccase enzyme production. *International Journal of Agriculture & Biology*.

[35] Naureen U., Arif R., Akram F., Shahid M. G., Saleem M. (2021). Genetic diversity analysis and in silico investigation of post-translational modifications of carboxypeptidase A1 (CpA1) in Sordaria fimicola. *International Journal of Agriculture and Biology*.

[36] Arif R., Bukhari S. H., Ishfaq M., Shahid M. G., Lee S. F., Saleem M. (2019). Genetic variation and post-translational modifications of cytochrome c oxidase-1 (COX1) in different strains of Sordaria fimicola. *International Journal of Agriculture and Biology*.

[37] Mobeen I., Arif R., Rasheed A., Akram F., Shahid M. G., Saleem M. (2020). Genetic and post-translational modification analysis of translational associated protein RKM4 in Sordaria fimicola. *International Journal of Agriculture and Biology*.

[38] Huang D., Li Z.-H., You D., Zhou Y., Ye B.-C. (2015). Lysine acetylproteome analysis suggests its roles in primary and secondary metabolism in Saccharopolyspora erythraea. *Applied Microbiology and Biotechnology*.

[39] Pan J., Ye Z., Cheng Z., Peng X., Wen L., Zhao F. (2014). Systematic analysis of the lysine acetylome in vibrio parahemolyticus. *Journal of Proteome Research*.

[40] Jones J. D., O'Connor C. D. (2011). Protein acetylation in prokaryotes. *Proteomics*.

[41] Pandey R., MuÈller A., Napoli C. A. (2002). Analysis of histone acetyltransferase and histone deacetylase families of Arabidopsis thaliana suggests functional diversification of chromatin modification among multicellular eukaryotes. *Nucleic Acids Research*.

[42] Polevoda B., Sherman F. (2003). N-terminal acetyltransferases and sequence requirements for N-terminal acetylation of eukaryotic proteins. *Journal of Molecular Biology*.

[43] Al-Rabia M. W., Mohamed G. A., Ibrahim S. R. M., Asfour H. Z. (2021). Anti-inflammatory ergosterol derivatives from the endophytic fungus Fusarium chlamydosporum. *Natural Product Research*.

[44] Bukhari S. H., Mobeen I., Naureen U. (2020). Analysis of genetic polymorphisms and post translational modifications of cytochrome C-1 in Sordaria fimicola. *International Journal of Agriculture and Biology*.

[45] Jaeken J. (2016). *Glycosylation and Its Disorders: General Overview*.

[46] Moremen K. W., Tiemeyer M., Nairn A. V. (2012). Vertebrate protein glycosylation: diversity, synthesis and function. *Nature Reviews Molecular Cell Biology*.

[47] Goettig P. (2016). Effects of glycosylation on the enzymatic activity and mechanisms of proteases. *International Journal of Molecular Sciences*.

[48] Lee L. Y., Hincapie M., Packer N., Baker M. S., Hancock W. S., Fanayan S. (2012). An optimized approach for enrichment of glycoproteins from cell culture lysates using native multi-lectin affinity chromatography. *Journal of Separation Science*.

[49] Pinho S. S., Reis C. A. (2015). Glycosylation in cancer: mechanisms and clinical implications. *Nature Reviews Cancer*.

[50] Boon L., Ugarte-Berzal E., Vandooren J., Opdenakker G. (2016). Glycosylation of matrix metalloproteases and tissue inhibitors: present state, challenges and opportunities. *Biochemical Journal*.

[51] Dufour A., Sampson N. S., Zucker S., Cao J. (2008). Role of the hemopexin domain of matrix metalloproteinases in cell migration. *Journal of Cellular Physiology*.

[52] Paik W. K., Paik D. C., Kim S. (2007). Historical review: the field of protein methylation. *Trends in Biochemical Sciences*.

[53] Herz H.-M., Garruss A., Shilatifard A. (2013). SET for life: biochemical activities and biological functions of SET domain- containing proteins. *Trends in Biochemical Sciences*.

[54] Guo A., Gu H., Zhou J. (2014). Immunoaffinity enrichment and mass spectrometry analysis of protein methylation. *Molecular & Cellular Proteomics*.

[55] Yagoub D., Hart-Smith G., Moecking J., Erce M. A., Wilkins M. R. (2015). Yeast proteins Gar1p, Nop1p, Npl3p, Nsr1p, and Rps2p are natively methylated and are substrates of the arginine methyltransferase Hmt1p. *Proteomics*.

[56] Ossareh-Nazari B., Gwizdek C., Dargemont C. (2001). Protein export from the nucleus. *Traffic*.

[57] Fischer U., Huber J., Boelens W. C., Mattajt L. W., Lührmann R. (1995). The HIV-1 Rev activation domain is a nuclear export signal that accesses an export pathway used by specific cellular RNAs. *Cell*.

[58] Wen W., Meinkotht J. L., Tsien R. Y., Taylor S. S. (1995). Identification of a signal for rapid export of proteins from the nucleus. *Cell*.

[59] La Cour T., Kiemer L., Mølgaard A., Gupta R., Skriver K., Brunak S. (2004). Analysis and prediction of leucine-rich nuclear export signals. *Protein Engineering Design and Selection*.

